# Participatory Art Activities Increase Salivary Oxytocin Secretion of ASD Children

**DOI:** 10.3390/brainsci10100680

**Published:** 2020-09-27

**Authors:** Sanae Tanaka, Aiko Komagome, Aya Iguchi-Sherry, Akiko Nagasaka, Teruko Yuhi, Haruhiro Higashida, Maki Rooksby, Mitsuru Kikuchi, Oko Arai, Kana Minami, Takahiro Tsuji, Chiharu Tsuji

**Affiliations:** 1 Division of Integrated Art and Sciences and Local Community Support, Research Center for Child Mental Development, Kanazawa University, Kanazawa 920-8640, Japan; tanakast@staff.kanazawa-u.ac.jp; 2 The COI Site, Tokyo University of the Arts Tokyo 110-8714, Japan; komagome.aiko@ms.geidai.ac.jp (A.K.); tanaka.oko@ms.geidai.ac.jp (O.A.); 3 Artlink Central, Scotland FK8 1EA, UK; ayaiguchi@icloud.com; 4 Department of Childhood Care and Education, Faculty of Social Work, Kinjo University, Hakusan 924-8511, Japan; ngsk-a4@kinjo.ac.jp; 5 Department of Basic Research on Social Recognition, Research Center for Child Mental Development, Kanazawa University, Kanazawa 920-8640, Japan; y-teruko@med.kanazawa-u.ac.jp (T.Y.); haruhiro@med.kanazawa-u.ac.jp (H.H.); minami-k@staff.kanazawa-u.ac.jp (K.M.); tsuji-t@u-fukui.ac.jp (T.T.); 6 Adverse Childhood Experiences (ACE) Lab, Institute of Health and Wellbeing, University of Glasgow, Glasgow G12 8RZ, UK; Maki.Rooksby@glasgow.ac.uk; 7 Social Brain in Action Lab, Institute of Neuroscience and Psychology, University of Glasgow, Glasgow G12 8QB, UK; 8 Department of Psychiatry and Neurobiology, Graduate School of Medical Science, Kanazawa University, Kanazawa 920-8640, Japan; mitsuruk@med.kanazawa-u.ac.jp; 9 Department of Health Development Nursing, Institute of Medical, Pharmaceutical and Health Sciences, Kanazawa University, Kanazawa 920-0942, Japan; 10 Department of Ophthalmology, Faculty of Medical Sciences, University of Fukui, Fukui 910-1193, Japan

**Keywords:** autism spectrum disorder, oxytocin, cortisol, group activity, stress, art

## Abstract

Autism spectrum disorder (ASD) occurs in 1 in 160 children worldwide. Individuals with ASD tend to be unique in the way that they comprehend themselves and others, as well as in the way that they interact and socialize, which can lead to challenges with social adaptation. There is currently no medication to improve the social deficit of children with ASD, and consequently, behavioral and complementary/alternative intervention plays an important role. In the present pilot study, we focused on the neuroendocrinological response to participatory art activities, which are known to have a positive effect on emotion, self-expression, sociability, and physical wellbeing. We collected saliva from 12 children with ASD and eight typically developed (TD) children before and after a visual art-based participatory art workshop to measure the levels of oxytocin, a neuropeptide involved in a wide range of social behaviors. We demonstrated that the rate of increase in salivary oxytocin following art activities in ASD children was significantly higher than that in TD children. In contrast, the change rate of salivary cortisol after participatory art activities was similar between the two groups. These results suggest that the beneficial effects of participatory art activities may be partially mediated by oxytocin release, and may have therapeutic potential for disorders involving social dysfunction.

## 1. Introduction

Autism spectrum disorder (ASD) is estimated to occur in 1 in 160 children worldwide [1]; ASD tends to begin in childhood and tends to persist for life. Deficits in social skills are the defining symptoms of ASD [2], which greatly impede the ability of individuals to function in community settings throughout their lives. Effective social interaction is essential for building friendships and avoiding unnecessary aversive interactions with peers during the early years, while communication with coworkers and customers is necessary for successful work performance in adults. Thus, social deficits are likely to lead ASD individuals to socially withdraw or isolate. Despite these serious consequences of social deficits in ASD, there is currently no medication to attenuate the impairment of social behaviors.

Moreover, in many cases, depression and anxiety accompany social deficits and other core symptoms of ASD such as repetitive behaviors and a narrow range of interests [2,3]. Therefore, numerous studies have focused on the hypothalamic–pituitary–adrenal (HPA) axis with regard to level of cortisol (CORT), which is frequently used as a stress biomarker [4]. The HPA axis is a highly regulated system that enables stress adaptation and diurnal rhythm, and CORT is secreted when the HPA axis is activated [5,6]. Previous studies have shown that in children with ASD, diurnal rhythm of CORT differs from that in typically developed (TD) children; in addition, children with ASD exhibit hyperresponsive CORT secretions in the social evaluative context or social situations [7]. Dysfunction of the HPA axis has been shown to negatively affect mental health in TD children and children with ASD [3,8]. Thus, management of stress in children with ASD may help decrease the risk of a concurrent mental disorder.

Recently, the therapeutic use of oxytocin (OT), which has a diverse role in mammalian social behaviors, has gained increasing attention. OT is a nonapeptide produced by the hypothalamic neurons in the brain. It is released to the bloodstream to reach peripheral organs or within the hypothalamic and other limbic regions of the brain [9]. The involvement of OT in social recognition, selective social bonding, attachment, anxiety, and stress coping has been demonstrated in a considerable amount of animal studies [9,10]. Indeed, mice that have a deficiency in OT release, such as OT null mice or CD38 knockout mice, are unable to recognize previously encountered conspecifics [11,12]. Furthermore, OT has been shown to be important for mate-bonding in monogamous prairie voles [13], as well as in parent-infant bonding in prairie voles, sheep, and primates [14,15]. The OT system is activated in stressful, challenging, and threatening conditions, and functions to facilitate stress coping [9]. OT has also been implicated in human emotions and social behaviors [14]; for instance, physical interaction between parents and infants increase endogenous OT in both the mother and father, and the increase in OT correlates with the level of affectionate behavior of the mother [15]. 

Exogenously administrated OT has been shown to modulate social behaviors, stress, and anxiety both in healthy individuals and in patients with neuropsychiatric disorders [16]. Moreover, intranasal administration of OT affects social perception, in that it improves discrimination of facial expressions [17,18] and increases the gaze to the eye region [19]. In addition, OT application has been previously shown to enhance feelings of trust [20] and generosity [21]. Thus, the peripheral application of OT has been considered a potentially effective therapeutic treatment for autism and schizophrenia; however, recent clinical trials have shown conflicting results [22]. Although this is in part due to the underpowered design of the trials, the use of exogenous OT as a therapeutic agent is questionable given it has low blood brain barrier permeability and metabolic stability in plasma [23]. Therefore, nonpharmacological interventions that increase endogenous OT are needed to ameliorate the social deficit of patients with ASD, especially those that can effectively stimulate the release of OT in the brain.

The World Health Organization (WHO) recently summarized how art-based interventions can help improve health and well-being, as well as contribute to the prevention of, and recovery from mental and physical illness [24]. Participatory art, in which participants engage in creative activities during social interaction, is known for its beneficial effects on physical health and emotional well-being [25,26]. In fact, the experience of participating in the creative process itself improves mental health conditions, thereby building resilience, boosting social and communicative skills, and improving self-confidence [26,27,28]. More broadly, such activities are included in social prescribing, an emerging intervention modality that has garnered interest and attention in the academic and clinical communities [29]. With participatory art, as well as social prescribing, the approach to treating various health ailments is holistic and practical, whereby activities such as art, exercising, and gardening are supervised by trained specialists, many of whom are nonclinicians [30]. Because ASD is a neurodevelopmental disorder for which no specific intervention has been established, such activities may provide valuable coping mechanisms across the life span for those affected and their families.

In the present study, we conducted the preliminary study to investigate whether the salivary OT level changes following participation in visual art-based activities in ASD and typically developed (TD) children. Because ASD individuals often struggle to adapt to changes or a new environment, we also measured the salivary CORT level, which is often used as a psychological stress biomarker. To the best of our knowledge, this is the first study to assess the neuroendocrinological changes in individuals who have taken part in participatory art activities.

## 2. Materials and Methods 

### 2.1. Participants

For this study, 10 TD children (aged 8–9 years) and 13 children (8–13 years) with ASD were recruited. All the children were volunteers; however, all children did not participate in all five sessions of visual art-based participatory workshops, and the data from children who participated less than twice were not used for analysis. Four boys and 4 girls, aged 8–9 years, who were TD and 11 boys and 1 girl, aged 8–13 years, who had ASD were analyzed (Table 1). ASD was diagnosed by children’s psychiatrists or pediatricians, who used criteria from the American Psychiatric Association’s Diagnostic and Statistical Manual of Mental Disorders, Fourth Edition, Text Revision (DSM-IV-TR) or Fifth Edition (DSM-V) or from the International Statistical Classification of Diseases and Related Health Problems, 10th edition (ICD-10). The evaluations included IQ testing, behavioral observation, and questionnaires; depending on the child, the Childhood Autism Rating Scale (CARS), Autism Diagnostic Observation Schedule (ADOS), Diagnostic Interview for Social and Communication Disorders (DISCO), or Pervasive Developmental Disorders Autism Society Japan Rating Scale (PARS) was used.

The participants’ parents were asked to respond to the Early Symptomatic Syndromes Eliciting Neurodevelopmental Clinical Examinations-Questionnaire (ESSENCE-Q) in order to screen the current status of TD children and children with ASD. The ESSENCE-Q is a brief screening questionnaire established specifically for the purpose of shortening the identification process of a wide variety of neurodevelopmental problems [31].

Among the children with ASD, 10 were enrolled in either regular or special needs classes at local schools in Japan. The other 2 children in this group were students at special support education schools.

### 2.2. Ethics Statement

The study was approved as a noninvasive medical study by Kanazawa University Graduate School of Medicine in 2018 (approval number #2790-2). The study was performed according to the Declaration of Helsinki and the Ethical Guidelines for Clinical Studies of the Ministry of Health, Labor and Welfare of Japan. After the children and their parents had been given a complete explanation of the study, they provided written informed consent. The participants were informed that they could choose not to supply their saliva on each occasion, even after agreeing to participate in the study.

### 2.3. Visual Art-Based Participatory Art Workshop

During the visual art-based participatory art workshops, participants created their own original stories and made characters and props with different kind of materials. Then participants used the Smoovie application on an iPad (Apple, Inc., Cupertino, CA, USA) to make their own movies with short stop-motion animation. The subjects of their stories varied from their everyday life to the objects that surrounded them; the theme of the project was fully participant led. The aim of this workshop was to let the participants express themselves as freely as possible and not to make them feel as if they were working on a school task or a job. We provided arts and crafts materials that stimulated different senses—paper (colored, textured, plain; different weights of paper), fabric (patterned, textured), tape (washi tape, colored tape), soft clay (colored, neutral), soft construction material (bubble wrap, wrapping material, cardboard), glue (hot glue gun, glue stick), paint, colored pens, and decorative material (sequins, gems, buttons).

The facilitators and supporters of the art workshop set up a comfortable venue, prepared the tools, explained how to use them, and provided a variety of materials. The art workshops conducted in this study were facilitated by a visual artist and supported by the art faculty, students from the University of the Arts, and a staff member who is licensed in special education. During the activity, the organizers interacted with the participants as and when appropriate and encouraged them throughout the creative processes of generating ideas and producing animations. 

### 2.4. Assessment

The visual art-based participatory art workshops were held five times between 22 July–10 August in 2018 and 2019. The workshops in 2018 were held from 14:30–16:30, while those in 2019 were held from 14:00–15:30. Sessions for TD children and ASD children were organized separately. The saliva samples were collected within 10 min of arrival, and just after the participatory art was finished. The percentage of salivary OT or CORT for each session was determined as (concentration after session/concentration before session) × 100. The total number of sessions analyzed before and after the art workshop and the percentage after the workshops for TD and ASD group were 33 and 42, respectively. The personal average percentage after the workshops was calculated as a mean percentage of all the sessions that each child participated (8 subjects for TD group and 12 subjects for ASD group). 

### 2.5. Saliva Collection and Analysis

The saliva samples (0.3–1.0 mL) were collected in a sterile 50 mL polypropylene tube (Greiner Bio-one Co. Ltd., Tokyo, Japan), and were immediately placed in ice for storage at −20 °C. Three to 5 days later, the samples were thawed and centrifuged twice at 4 °C at 1500× *g* for 10 min. The samples were divided into 1.5-mL microtubes, each containing 100 µL, and kept again at −80 °C until required for the assay. The salivary OT level was measured using a 96-plate commercial OT-ELISA kit (Enzo Life Sciences, Farmingdale, NY, USA), as described previously [32,33,34]. The measurements were performed in duplicate. Samples (100 µL) without fractionation were treated according to the manufacturer’s instructions. The optical density of the samples and standards was measured at wavelengths of 405 nm by a microplate reader (Bio-Rad, Richmond, CA, USA). The salivary CORT level was measured using a cortisol enzyme immunoassay kit (Salimetrics, State College, PA, USA) as previously described [35]. Samples (25 µL) were treated according to the manufacturer’s instructions. The optical density of the samples and standards were measured at wavelengths of 450 nm by a microplate reader (Bio-Rad, Richmond, CA, USA). Measurements were performed in duplicate. Sample concentrations were calculated according to the relevant standard curve. The intraassay and interassay coefficients were <12.4% and <12.1%, respectively.

### 2.6. Statistical Analysis

Statistical analysis was performed using Prism 8 software (GraphPad Software Inc., San Diego, CA, USA). Wilcoxon rank-sum tests were used to compare the levels of salivary OT or CORT of TD children with children with ASD before and after each visual art-based workshop session. The Mann–Whitney *U* test was used to compare the average percentage of OT or CORT of TD children with children with ASD after each session. Two-tailed student’s *t* test was used to assess the personal average percentage of salivary OT or CORT after each session in which a child participated. All data were calculated as means ± standard errors of the means. In all analyses, *p* < 0.05 was considered statistically significant.

## 3. Results

**Figure 1:**
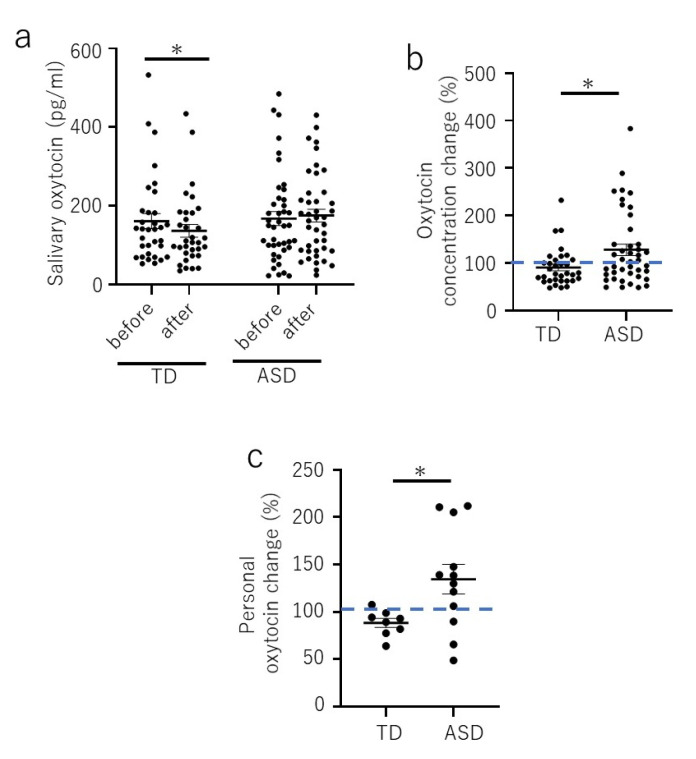
The salivary oxytocin levels before and after participatory art workshops. (**a**). Salivary oxytocin level before and after each session in the typically developed (TD) children and those with autism spectrum disorder (ASD). Note that not all children attended all five sessions of the art workshop (total number of sessions analyzed: 33 for the TD children and 42 for the children with ASD). (**b**). Percentage of salivary oxytocin in the TD children and those with ASD after each session. Note that not all children attended all five sessions of the art workshop (total number of sessions analyzed—33 for the TD children and 42 for the children with ASD). (**c**). Personal average percentage of salivary oxytocin level after each session in the TD and ASD groups (number of children assessed: TD, *n* = 8; ASD, *n* = 12). Data are mean ± sem. * *p* < 0.05.

The salivary OT level after each session of visual art-based participatory art workshop showed a significant decrease in the TD group (before, 161.5 ± 19.4 pg/mL, after 136.6 ± 16.0 pg/mL; *p* = 0.015) (Figure 1a), whereas there was no significant difference in the ASD group (before 167.9 ± 17.9 pg/mL, after 175.5 ± 16.4 pg/mL; *p* = 0.880) (Figure 1a). However, the average percentage of salivary OT after each session of the ASD group was significantly higher than that of the TD group (TD, 90.8% ± 6.9%; ASD, 127.9% ± 11.8%; *p* = 0.024) (Figure 1b). Also, the personal average percentage of salivary OT after each participated session of the ASD group was significantly higher than that of the TD group (TD, 88.4% ± 4.8%; ASD, 134.5% ± 15.6%; *p* = 0.03) (Figure 1c).

**Figure 2:**
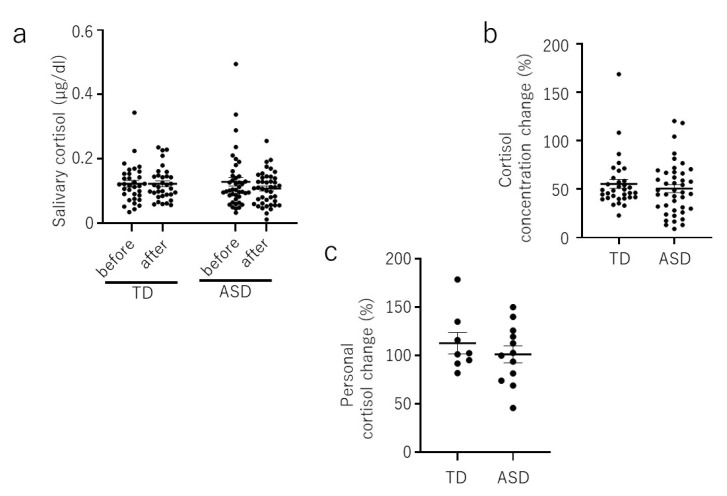
The salivary cortisol level before and after participatory art workshop. (**a**). Salivary cortisol level before and after each session in the TD and ASD groups. Note that not all children attended all five sessions of the art workshop (total number of sessions analyzed—33 for the TD children and 42 for the children with ASD). (**b**). Percentage of salivary cortisol after each session in the TD and ASD groups. Note that not all children attended all five sessions of the art workshop (total number of sessions analyzed—33 for the TD children and 42 for the children with ASD). (**c**). Personal average percentage of salivary cortisol level after each session in the TD and ASD groups (number of children assessed: TD, *n* = 8; ASD, *n* = 12). Data are mean ± sem.

The concentrations of salivary cortisol were not significantly different before and after the participatory art workshop in either the TD or ASD group (Figure 2a). No significant difference was observed between the TD and ASD groups with regards to the average percentage after each session, and/or in the average percentage of personal salivary CORT after each session (Figure 2b,c). 

## 4. Discussion

The current trial was a pioneering study to assess salivary OT and CORT concentrations in ASD and TD children in response to a positive social activity, participatory arts. Although we could not detect a statistical difference in OT levels after each session in the ASD group, the average percentage of salivary OT after each session, and the personal average percentage of salivary OT after each session were increased in ASD children. However, no significant difference in the cortisol level was detected before and after participatory art activities in either the ASD or TD group. Since the cortisol level did not change significantly during the participatory art activities, we suggest that OT is released as a result of positive social activities, rather than a response to stress. 

Because all the children participated voluntarily in the visual art-based participatory art workshop, the TD children were expected not to feel anxious or stressed about their participation, and we initially assumed that their OT levels would increase after participatory art activities. However, the OT level decreased instead. As the CORT level in TD children did not change significantly, the decrease in the OT level may not be a result of the stress response. Because both groups of children enjoyed the art activities, the results suggest that the TD children may have felt driven to complete their artwork, which corresponded with a decrease in the OT level afterwards. A decrease in OT levels has been suggested to reflect a reduction in social qualities of attention or, more broadly, in social interaction [36]. In our case, the decrease in the TD group may correspond to a shift in attention upon completion of their work. The children with ASD, on the other hand, required the experience to form a part of their routine over the course of the workshop attendance and may have gained more pleasure out of the process of creativity. It has been reported that attention systems are disrupted and atypical in children with ASD [37]. It is possible that our participants with ASD required more sessions than the TD children in order to understand the structure of the workshops and to focus on their creative activities. Alternatively, there might have been an increase in the OT level in TD children during the activities, but the time course may have differed from that observed in the children with ASD. Larger sample sizes and further follow up will allow a better understanding of the process.

Our preliminary study had several limitations. First, the study was designed to examine the immediate OT response of participatory art activity. Although the percentage change of OT level was increased in ASD children, we failed to show a statistical difference in the salivary OT level before and after each of the workshop due to the modest sample size and the large variance in the OT concentration. Therefore, it is necessary to conduct a further trial with a larger sample size of ASD children to obtain reliable data. Second, we did not include any psychological, emotional, or behavioral measurement that may be relevant to the OT response. As shown in Figure 1c, there is a diverse variability in the personal percentage change in ASD children compared to the TD children; as a result, we need to conduct a further study to elucidate the correlation between the traits of children and the OT responsiveness to participatory art activities. Third, it is worth pointing out that our study did not include a control condition in order to disentangle the potential effect of participatory art from other more generic activities involving crafting or animation making. Such a control could have simply involved being in the premise for the workshop sessions, as in a wait-list group. However, each workshop typically required the entire duration of the children’s visit in terms of staff cover and their families who need to fit in taking and collecting their children. Staffs and participants were all volunteers and we faced several practical difficulties which prevented from adding this important aspect to our design this time. We will be mindful for exploring options for adding the control in our future work.

The WHO recently summarized how art interventions can help improve health and well-being, and can help to prevent both mental and physical illness [24]. They also stated that while art is conceptually difficult to define, it is important to verify the effectiveness of art. Indeed, although a wide variety of studies use diverse methodologies, these are often based on psychological tests or subjective feedback. Our study aimed to capture the fluctuations in the neuroendocrine response to participatory art activities. In the future study, we will investigate the association of the neuroendocrine response and the traits of children in order to elucidate for whom and in what aspect participatory art activities can be effective. We will also evaluate the effectiveness of participatory arts activities in the long term. Further, experience in multi-sensory processing has been showing some promising results in children with autism spectrum disorders [38]. Given that crafting and creative activities are familiar and popular in typical as well as atypical development, it would be an important agenda to study the therapeutic role of multi-sensory experiences involved in activities such as participatory art. Ultimately, we would like to utilize our research findings related to the use of neuroendocrine responses in combination with psychological, emotional, or behavioral measurement to establish a method to verify the effectiveness of participatory art activities. 

In the current study, ELISA was used to measure the saliva OT concentrations; however, with this technique, there remains the issue of antibody specificity. The antibody may not only detect the free form of the target analyte, but different forms, such as precursors and metabolites, or a form within other protein complexes. These phenomena may partially explain the differences in values observed between different ELISA kits, as well as between ELISA and HPLC-MS or radio immunoassay. Indeed, our detected OT concentration range is one digit higher than that reported by other studies [15,39,40]. However, since we have detected the relative change in salivary OT in the context of physical interaction and/or in social interaction [32,34,41], although the values may not be reflecting the absolute physiological value of free OT, the relative change in monitored values are likely to be comparable.

In conclusion, the current experiment was designed to assess salivary OT and CORT concentrations in ASD and TD children in response to participation in visual art-based activities, since art is known to improve mental and physical health and overall quality of life. We demonstrated increased OT release after participatory art activities that was unrelated to the stress response. Thus, our case study is the first to show the biological link between the social experience of participatory art and its positive psychosocial effects. 

## Figures and Tables

**Table 1:** Age, mean scores of Early Symptomatic Syndromes Eliciting Neurodevelopmental Clinical Examinations-Questionnaire (ESSENCE-Q) and education for each group of children.

	TD	ASD
Numbers	8 (male: 4, female: 4)	12 (male: 11, female: 1)
Age	96–119 months	113–162 months
	mean: 107, SD: 6.9	mean: 135, SD: 16.7
ESSENCE-Q	Yes: 0.4, Maybe/A Little: 0.1/0.3	Yes: 2.83, Maybe/A Little: 1.58/2.83
Education	regular class	regular or special needs classes or special education support classes
